# Bezafibrate Improves Mitochondrial Fission and Function in DNM1L-Deficient Patient Cells

**DOI:** 10.3390/cells9020301

**Published:** 2020-01-27

**Authors:** Liza Douiev, Ruth Sheffer, Gabriella Horvath, Ann Saada

**Affiliations:** 1 Department of Genetic and Metabolic Diseases and Jacques Roboh Department of Genetic Research, Hadassah Medical Center, Jerusalem 9112001, Israel; RuthSh@hadassah.org.il; 2 Division of Biochemical Diseases, Department of Pediatrics, University of British Columbia, Vancouver, BC BC V6H 3V4, Canada; Gabriella.Horvath@cw.bc.ca; 3 Faculty of Medicine, Hebrew University of Jerusalem, Jerusalem 9112001, Israel

**Keywords:** DNM1L, Drp1, mitochondrial disease, mitochondrial fission-fusion, bezafibrate, fibroblast

## Abstract

Mitochondria are involved in many cellular processes and their main role is cellular energy production. They constantly undergo fission and fusion, and these counteracting processes are under strict balance. The cytosolic dynamin-related protein 1, Drp1, or dynamin-1-like protein (DNM1L) mediates mitochondrial and peroxisomal division. Defects in the *DNM1L* gene result in a complex neurodevelopmental disorder with heterogeneous symptoms affecting multiple organ systems. Currently there is no curative treatment available for this condition. We have previously described a patient with a de novo heterozygous c.1084G>A (p.G362S) *DNM1L* mutation and studied the effects of a small molecule, bezafibrate, on mitochondrial functions in this patient’s fibroblasts compared to controls. Bezafibrate normalized growth on glucose-free medium, as well as ATP production and oxygen consumption. It improved mitochondrial morphology in the patient’s fibroblasts, although causing a mild increase in ROS production at the same time. A human foreskin fibroblast cell line overexpressing the p.G362S mutation showed aberrant mitochondrial morphology, which normalized in the presence of bezafibrate. Further studies would be needed to show the consistency of the response to bezafibrate, possibly using fibroblasts from patients with different mutations in *DNM1L*, and this treatment should be confirmed in clinical trials. However, taking into account the favorable effects in our study, we suggest that bezafibrate could be offered as a treatment option for patients with certain *DNM1L* mutations.

## 1. Introduction

Mitochondria are cytosolic double-membrane-bound organelles found in nucleated eukaryotic cells. They are involved in many cellular processes, including the regulation of cell proliferation and differentiation, intracellular calcium homeostasis, regulation of cell death, among others. However, their major role is cellular energy (ATP) production via oxidative phosphorylation (OXPHOS) [1]. Mitochondria are highly dynamic organelles, constantly undergoing fission and fusion. Their number, shape and location undergo changes throughout the cell cycle depending also on different environmental conditions and energy status. This process is mediated by a highly regulated balance between the counteracting processes of fission versus fusion [2,3,4,5,6,7].

Symptoms of mitochondrial disorders affecting OXPHOS are heterogeneous, affecting most of the organ systems in the body, and include muscle weakness, neurological deficits (including seizures), vision or hearing loss, poor growth, and involvement of other organs such as gastrointestinal, cardiac, or respiratory systems. These symptoms can present at any age, but if presenting early, usually imply a much more severe disease course, in many cases being fatal in infancy or early childhood [1]. Defects in mitochondrial genes encoding proteins involved in mitochondrial dynamics proteins, causing abnormal fission or fusion, can also affect OXPHOS and result in clinical disease. Among these, mutations in the gene encoding dynamin-1-like protein, *DNM1L*, are increasingly associated with human diseases. Since the first patient with a mutation in the *DNM1L* gene presenting with lethal epileptic encephalopathy was described by Waterham and colleagues in 2007 [8], there have been several case reports published. As of today, there are over 18 patients described, most of them having heterozygous de novo variants in the middle domain, which were shown to have a negative dominant effect. Others have variants in the GTPase head domain. The clinical phenotypes vary between neonatal hypotonia, microcephaly, apnea, persistent elevation of lactate, focal, progressing into generalized status epilepticus, with devastating impaired neurological outcome, and death early in infancy or childhood. Some other patients had a relatively normal early development then developed severe epileptic encephalopathy with intractable seizures and regression after an intercurrent illness, infection, or vaccination [9,10,11,12,13,14,15,16,17,18,19,20].

DNM1L is the human analogue to Drp1, a member of the dynamin superfamily of cytoplasmic GTPases. It is an evolutionarily conserved protein that mediates mitochondrial and peroxisomal division, and it is also involved in apoptotic regulation. Drp1 regulates mitochondrial fission through assembly of fission foci and distribution of mitochondrial tubules through the cytoplasm, and this is essential for normal mitochondrial function and turnover. Drp1 contains three domains prototypical of the dynamin-like GTPase class, with an N-terminal GTPase head, a middle-domain (MD), and a C-terminal GTPase effector domain (GED), as well as a non-conserved variable domain (VD). Drp1 is recruited to the mitochondrial outer membrane upon fission triggering. It self-assembles into a fission complex that forms a ring-like structure that constricts the organelle, thus providing the force to produce a local fission event. The mechanism of initial recruitment of Drp1 to the outer mitochondrial membrane is a rate-limiting step of mitochondrial fission. A shift in the mitochondrial fusion and fission equilibrium can drive the mitochondria to elongate or fragment in response to certain cellular demands, suggesting an intricate relationship between mitochondrial morphology and function. Consequences of the blockade of mitochondrial fission are—abnormal distribution of mitochondrial reactive oxygen species (ROS) generation, accumulation of polyubiquinated mitochondria, and loss of respiratory function [21,22,23,24,25], reviewed in [26,27].

Despite major advances in the scientific and medical community’s ability to diagnose and identify the genetic causes for mitochondrial diseases, treatment remains a major challenge. Generally, treatment has been supportive and symptomatic with little effect on overall outcome of the disease. Several therapeutic approaches have been proposed and investigated over the past years. The administration of a small-molecule-based approach is one of them. The use of small molecules aims to improve mitochondrial function, such as respiratory efficiency, ATP production, and reduction of the free radicals, among others. These small molecules are divided into several groups and include antioxidants, cofactors, mitochondrial biogenesis promoting factors, and others [28,29,30,31]. One such molecule is bezafibrate, which is considered as a peroxisome proliferator-activated receptor alpha (PPARα) agonist. PPARs are ligand-activated transcription factors, which upon activation form heterodimers with the retinoid X receptor (RXR). This heterodimer binds to a specific recognition sequence that is located within a gene regulatory region of PPAR-target genes. The PPAR family is composed of three isoforms. Each has a variety of target genes and exhibits different tissue distribution. Some of their target genes are nuclear-encoded respiratory chain (RC) genes, and others are mitochondrial biogenesis and fatty acid oxidation genes. The molecular mechanisms underlying bezafibrate effects on mitochondrial function and biogenesis are partially explained by its ability to upregulate the expression of nuclear-encoded mitochondrial genes [31,32].

The main goal of the current study was to examine the effect of bezafibrate on mitochondrial function in fibroblasts derived from a patient carrying a dominant negative *DNM1L* mutation. Our findings were corroborated in a fibroblast cell line overexpressing the same mutation. Our results suggest a possible therapeutic intervention for patients that carry mutations in this fission gene. 

## 2. Materials and Methods

### 2.1. Materials

Cell culture media and solutions were obtained from Biological Industries (Kibbutz Beit Ha’emek, Israel). Fluorescent mitochondrial dyes were purchased from Invitrogen (Carlsbad, CA, USA), H_2_DCFDA from Biotium (Hayward, CA, USA), and the ATPlite^TM^ was from Perkin Elmer (Waltham, MA, USA). Other reagents were purchased from Sigma-Aldrich (Rehovot, Israel) at the highest purity available.

### 2.2. Cell Culture

Previously established primary skin fibroblast cell lines available for the study were derived from the patient harboring the de novo heterozygous c.1084G>A (p.G362S) mutation [10] and controls. All subjects gave their informed onset for inclusion before they participated in the study conducted in accordance with the Declaration of Helsinki, and the protocol was approved by the Ethics Committee of Hebrew University-Hadassah Medical School #0485-09; 15.12014. Cells were cultured in a permissive high-glucose-DMEM (Dulbecco’s Modified Eagle Medium) medium containing 4.5 g/L glucose supplemented with 15% fetal calf serum (FCS), 1% penicillin–streptomycin, 365 μg/mL L-glutamine, 110 μg/mL pyruvate, and 50 μg/mL uridine at 37 °C, 5% CO_2_. Cell growth was also evaluated in a restrictive glucose-free DMEM medium supplemented with 10% dialyzed FCS and 5 mM galactose. To evaluate bezafibrate replaced with a fresh medium with or without 100 µM bezafibrate (200 mM stock solution in DMSO (Dimethyl Sulfoxide), stored at −20 °C) for 72 h prior to assessments. Cell viability was assessed by trypan blue exclusion.

### 2.3. Assays in Microtiter Wells

Assays were performed in microtiter wells, as we have previously described [33,34,35]. A total of 3000 viable cells/well were seeded overnight and replaced with new permissive or restrictive medium, in the presence or absence of bezafibrate. Cell content, ATP production, mitochondrial membrane potential (MMP), and reactive oxygen species (ROS) were evaluated as follows—cell content was measured by a colorimetric method using methylene blue (MB), which we have previously shown to reflect cell count [33,34]. Briefly, cells were fixed with 0.5% gluteraldehyde for 10 min, rinsed with double-distilled water, stained with 1% methylene blue in 0.1 M borate buffer pH 8.5 for 1 h, rinsed with water, and allowed to dry. The dye was extracted from the cells with 0.1 N HCl at 37 °C for 1 h then measured at A620 nm. The results are presented as growth in glucose-free medium divided by average cellular growth in high-glucose medium.

ATP production was measured by luciferin-luciferase, in digitonin-permeabilized cells in the presence of glutamate and malate, as we have previously described [35]. Briefly, cells were rinsed in PBS and incubated for 20 min at 37 °C in 50 μL assay buffer containing 150 mM KCl, 10 mM potassium phosphate buffer (pH 7.4), 25 mM Tris (pH 7.4), 2 mM EDTA, 0.025% fatty-acid-free bovine serum albumin (BSA), 40 μg/mL digitonin, 5 mM glutamate, and 1 mM malate. After incubation, ATP content was calculated as relative luminescence (RLU) by the ATPlite^TM^ luminescence assay system according to the manufacturers protocol (Perkin Elmer, Waltham, MA, USA).

Mitochondrial membrane potential (MMP) was estimated using double staining with MitoTracker Green (MTG) and tetramethylrhodamine ethyl ester (TMRE) (Molecular Probes, Eugene, OR, USA) [34,35]. Briefly, MTG was added to the existing medium to a final concentration of 200 nM and the cells were incubated for 45 min at 37 °C, 5% CO_2_. TMRE was added successively to the existing medium to a final concentration of 50 nM and the cells were incubated for an additional 45 min at 37 °C, 5% CO_2_. Medium was removed and, after rinsing once with PBS, replaced with 100 mL PBS. The plate was read at 37 °C, λex 485 nm, λem 528 nm (MTG), λex 485 nm, and λem 590 nm (TMRE).

Intracellular ROS production was measured in a fluorometric assay using 2′,7′-dichlorodihydrofluorescein diacetate (H_2_DCFDA) (Biotium, Harvard, CA, USA), as we previously described [33]. Briefly, growth medium was removed and replaced by 100 μL/well of 10 µM H_2_DCFDA in PBS-Ca^2+^ Mg^2+^ (PBS containing 0.9 mM calcium chloride and 0.5 mM magnesium chloride) and the plates were incubated for 20 min at 37 °C, 5% CO_2_. After removal of H_2_DCFDA, the ROS production was monitored for 20 min in 100 μL PBS-Ca^2+^ Mg^2+^ at λex 485 nm and λem 520 nm.

All values were normalized to cell growth as measured by MB in parallel wells for each separate experiment. Results are presented as mean ± SEM of experiments performed in triplicate wells on at least three separate occasions. Readings were obtained with a Synergy HT microplate reader instrument. Statistical analysis was made by Student’s *t*-test (IMB-SPSS 20). A *p*-value of <0.05 was considered statistically significant.

### 2.4. Microscopy

Mitochondrial morphology was visualized using MitoTracker Red CM-H2XRos (MTR)/A total of 3 × 10^5^ fibroblasts were seeded on 35 mm glass-bottom tissue culture plates in high-glucose medium and incubated with or without bezafibrate at 37 °C, 5% CO_2_. Subsequently, the cells were incubated with MTR (2 μM) for 30 min at 37 °C, 5% CO_2_ in the dark. Occasionally nuclei were stained with NucBlue (Thermo Fisher Scientific, Invitrogen, Waltham, MA, USA). The cells were visualized live by fluorescent, confocal microscopy (Olympus FV300) and mitochondrial length estimation was manually performed on at least 70 cells on two different occasions (double-blind by two different people) by measuring the length of cellular mitochondria relative to untreated controls using ImageJ software http://imagej.nih.gov/ij/.

### 2.5. Oxygen Consumption

Oxygen consumption was measured essentially as we have previously described [35] in the absence and presence of bezafibrate in the medium 72 h prior to measurement. Oxygen consumption rate (OCR) was measured using an XF24 extracellular flux analyzer (Seahorse Biosciences, North Billeric, MA, USA). Fibroblasts were seeded in 20,000 cells/well on an XF24-well plate in 0.3 mL in the absence or presence of 100 µM Bezafibrate. After 72 h, the growth medium was changed to a medium with the same composition as GLU, but with unbuffered DMEM (Seahorse Biosciences, North Billeric, MA, USA) and the plate was equilibrated at 37 °C for 1 h before the measurements. After 10 min of basal OCR and extracellular acidification rate (ECAR) measurements, carbonyl cyanide-4-(trifluoromethoxy)phenylhydrazone (FCCP) was injected to reach a final concentration of 5 μM and the maximal OCR was measured. Background OCR was measured after injection of rotenone and antimycin to a final concentration of 3 μM each. OCR and ECAR were calculated per cell count (trypan blue exclusion).

### 2.6. Overexpression of the Mutant Gene

Transformation of human foreskin fibroblasts (HFF-1 ATCC) was performed overnight with a custom-made expression vector pRP[Exp]-CMV>ORF: IRES:EGFP containing the pG362S *DNM1L* mutation or with a control vector pRP[Exp]-CMV>EGFP lacking the insert (Cyagen Biosciences, Sunnyvale, CA, USA) using Lipofectamine 2000 (Thermo Fisher Scientific, Invitrogen, US) in an Opti-MEM medium (Thermo Fisher Scientific, Waltham, MA, USA). Upon 72 h post-transfection, cells were grown in fresh medium in the presence of 100 µM bezafibrate or vehicle (DMSO). After an additional 72 h, the cells were stained with MTR and those expressing GFP (~80% of the cells) were examined by confocal fluorescence microscopy (cell were visualized live by Spinning Disc confocal microscopy, Nikon at A488 nm (GFP) and A580 nm (MTR).

## 3. Results

### 3.1. Bezafibrate Normalizes Growth, ATP Production, and Oxygen Consumption in Patient’s Fibroblasts

**Figure 1:**
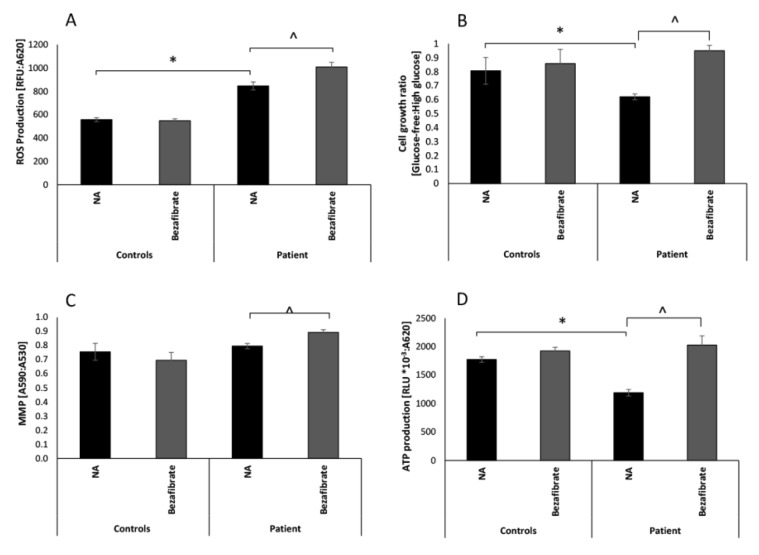
Patient and control fibroblasts’ growth, ATP production, mitochondrial membrane potential, and reactive oxygen species (ROS) production. Patient and control (n = 5) fibroblasts were seeded on 96-well plates and incubated for 72 h without additive (NA) or in the presence of 100 µM bezafibrate. Intracellular ROS production (**A**) was measured using fluorometry with H_2_DCFDA. Cell growth (**B**) was measured by methylene blue (MB) and presented as the ratio between cell growth in glucose-free and growth in high-glucose. Mitochondrial membrane potential (MMP) (**C**) was measured using double staining with MitoTracker Green (MTG) and tetramethylrhodamine ethyl ester (TMRE). ATP production from glutamate and malate (**D**) was measured by luciferin-luciferase luminescence. Values are presented as normalized mean ± SEM; * *p* < 0.05 patient compared controls in the corresponding medium; ^ *p* < 0.05 bezafibrate-treated compared to NA.

We have previously reported functional OXPHOS deficits and aberrant mitochondrial morphology in the DNM1L patient’s fibroblasts, thus we set out to evaluate the effect of bezafibrate on several OXPHOS parameters [10]. To evaluate growth, patient and control fibroblasts were incubated in high-glucose (permissive) and a glucose-free, galactose containing (restrictive) media. Notably, cells grown under permissive conditions derive most of their energy from glucose via glycolysis, whereas growth in the restrictive medium forces cells to rely on mitochondrial oxidation of fatty acids and glutamate via the OXPHOS. Accordingly, cells with OXPHOS defects exhibit defective growth (62% of control) in glucose-free medium [36]. This was observed also in the present case, as growth ratio in glucose-free compared to high-glucose media was significantly lower in the patient’s fibroblasts (0.62) compared to control fibroblasts (0.81) (Figure 1B). The patient’s fibroblasts supplemented with bezafibrate showed significantly increased growth ratio (0.95), which was 117% and 110% of untreated or treated control cells, respectively. This was even a higher ratio than in control cells where the mild effect was not statistically significant. We verified our preliminary results [10], confirming that the patient’s fibroblasts produced decreased levels (66% of controls) of ATP (Figure 1D). Supplementation with bezafibrate induced a significant increase (170% relative to no additive), thereby restoring (114% of controls) ATP production. Bezafibrate mildly increased the ATP production also in controls albeit not in a statistically significant way.

Although previously we did not detect any significant decrease in MMP, we still evaluated this parameter to evaluate the effect of bezafibrate and observed a minor increase (105%), which was not significantly different from the control cells (Figure 1C). A slight elevation of MMP (112%) was observed upon bezafibrate treatment. Bezafibrate was not able to ameliorate the significantly elevated basal ROS production in patient cells (Figure 1A). Moreover, the supplementation slightly increased ROS production by 20% in the patient’s cells but with no apparent effect on controls. The elevated ROS in the patient’s fibroblasts could be a secondary outcome of the increased mitochondrial content (46%) (Figure S1).

Maximal oxygen consumption in the patient’s fibroblasts was decreased to 68% of controls at baseline and normalized (105%) with bezafibrate treatment. The ratio of basal to maximal oxygen consumption did not differ between patient and control (2.5), treated or untreated (2.6), thus there was no evidence of uncoupling. Bezafibrate also increased extracellular acidification (ECAR) rate indicating elevated glycolysis as well (Figure S1).

Additionally, we examined the effect of 20 µM bezafibrate, however the results were not statistically significant. We also attempted to increase the bezafibrate to 200 µM, however at this concentration the vehicle DMSO control sample showed a mild negative effect.

### 3.2. Bezafibrate Improves Mitochondrial Morphology in Patients’ Fibroblasts

**Figure 2:**
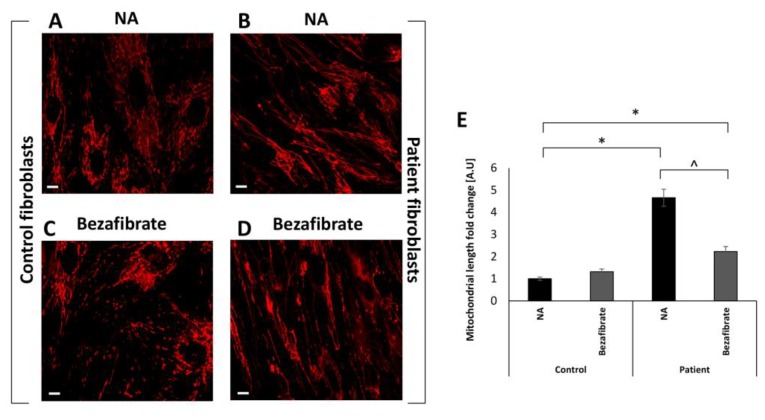
Mitochondrial morphology. Control (**A**,**C**) and the patient’s fibroblasts (**B**,**D**) were seeded in 35 mm glass-bottom tissue culture plates. The following day, the medium was replaced with fresh high-glucose medium without (**A**,**B**) additive (NA) or in the presence of bezafibrate (**C**,**D**) and incubated for 72 h. Mitochondrial morphology was visualized by MitoTracker Red CM-H2XRos. Relative mitochondrial length was calculated and depicted (**E**) using ImageJ software. Mean normalized to control no additive ± SEM; * *p* < 0.05 compared to NA control; ^ *p* < 0.05 compared to same cell with no additive. Scale bar = 10 µM.

As we have previously shown [10], mitochondrial morphology in patient fibroblasts is aberrant with markedly elongated mitochondria compared to normal fibroblasts grown under the same conditions (Figure 2A,B). Evidently, bezafibrate had an apparent significant effect on mitochondrial morphology, which was mainly expressed by a less elongated phenotype in the patient’s fibroblasts while controls were largely unaffected (Figure 2C,D). The histogram in Figure 2E depicts the quantitative estimation of average mitochondrial length, showing that the patient’s mitochondria were more than four times longer relative to controls. Bezafibrate markedly shortened mitochondrial length in the patient’s cells to less than half of the untreated cells, although remaining more elongated than controls.

### 3.3. Bezafibrate Improves Mitochondrial Morphology, Content, and Viability in Fibroblasts Overexpressing the p.G362S DNM1L Mutation

**Figure 3:**
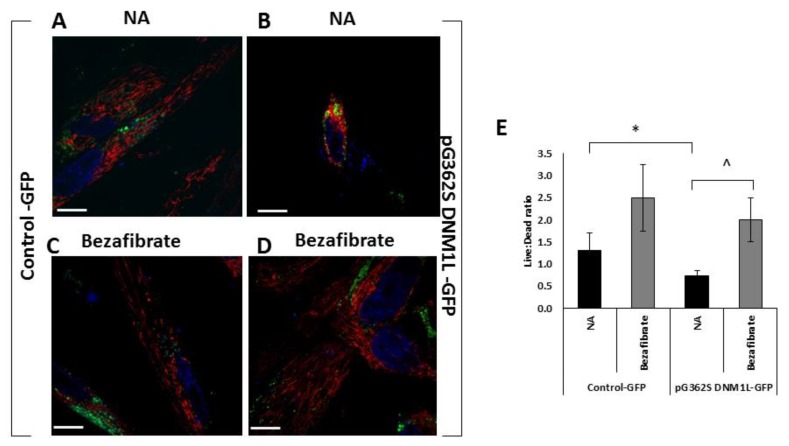
Mitochondrial morphology and viability of human foreskin fibroblast (HFF-1) cells expressing the p.G362S DNM1L mutation. Human foreskin fibroblasts, transformed with a control plasmid expressing GFP (**A**,**C**) or a plasmid expressing GFP and p.G362S DNM1L mutation (**B**,**D**), were seeded in 35mm glass-bottom tissue culture plates 72 h post-transformation. The following day, the medium was replaced with fresh high-glucose medium without (**A**,**B**) additive (NA) or in the presence of bezafibrate (**C**,**D**) and incubated for 72 h. Mitochondrial morphology was visualized by MitoTracker Red CM-H2XRos (red) and was examined in cells expressing GFP (green) by fluorescence microscopy. Nuclei were stained in blue. Scale bar = 10 µM. In parallel experiments, cell viability was measured by trypan blue and the results are depicted in the histogram (**E**) ± SEM; * *p* < 0.05 compared to NA control; ^ *p* < 0.05 compared to the same cell with no additive.

Transformation of HFF-1 cells, with plasmid expression carrying the p.G362S DNM1L mutation, demonstrated smaller cell size (56%) with aberrant mitochondrial morphology showing perinuclear, aggregated mitochondria compared to the control vector (Figure 3A,B). This is also in accordance with our previous findings [10] and our assumption that fission is exceedingly impaired, hindering mitochondria to elongate and separate from each other. The mitochondrial morphology normalized in the presence of bezafibrate although it was not possible to calculate mitochondrial length due to the aggregation (Figure 3C,D). Mitochondrial content, estimated by MTR fluorescence, was slightly increased (120% of untreated cells), but not statistically significant, both in control and in the mutant cells.

As could be expected, transformation did affect viability. However, the cells carrying the mutant gene were significantly adversely affected 53% (0.7 live:dead ratio) compared to controls (1.3 live:dead ratio) (Figure 3E). Interestingly, bezafibrate had a generally positive effect on viability in both control (190% of untreated) and more so in the mutant cells (280%).

## 4. Discussion, Limitations, and Conclusions

### 4.1. Discussion

The mitochondrion is a highly dynamic organelle that constantly fuses and divides. These two processes are crucial for proper mitochondrial function and mutation in components regulating these processes cause mitochondrial diseases, with impaired mitochondrial function and pathological metabolism and signaling reviewed in [26,27,37]. The mutation of our patient, located in *DNM1L,* causing a disruption in the fission process, leads to several functional consequences expressed in fibroblasts, including cell growth on glucose-free media, ROS and ATP production, the mitochondrial morphology, [10] and present results. Thus, these cells are suitable as a model system for evaluating the effect of small molecules on an individual basis, as we and others have done in the past [38,39]. In the current study, where we specifically aimed to evaluate the effect of bezafibrate while screening several other small molecules, our preliminary results showed to be promising (previously unpublished), while other molecules were less effective [40].

Bezafibrate is a lipid-lowering fibrate drug which also improves insulin sensitivity. Its effects are attributed to the activation of PPARs, which play essential roles in metabolism. Being a pan-PPAR agonist, bezafibrate also activates the PPAR–peroxisome proliferator-activated receptor gamma coactivator 1-alpha (PGC-1α) axis. PGC-1α overexpression is known to increase mitochondrial biogenesis and oxidative function by coordinating the transcription of genes encoded by the nuclear and mitochondrial genomes via coactivation of several important transcription factors involved in energy metabolism, such as estrogen-related receptors (ERRs), the nuclear respiratory factors 1 and 2 (NRF1/2), and mitochondrial transcription factor ATFAM reviewed by Villena [41]. As such, bezafibrate has been proposed as a treatment option for diseases involving mitochondrial function. Indeed, data obtained in fibroblasts from mitochondrial fatty acid oxidation defects treated with bezafibrate showed upregulation of the expression of the affected gene and improved fatty oxidation flux. Moreover, bezafibrate exerted a stimulatory effect on the activity of respiratory chain complexes and on cellular oxygen consumption in fibroblasts from respiratory chain defective cell lines that exhibited some level of residual RC function, as reviewed by Djouadi and Bastin [31].

To the best of our knowledge, this compound has never been investigated in the context of a mitochondrial fission defect. Our results show a positive effect of bezafibrate on a *DNM1L* defect, with respect to growth in glucose-free medium and a markedly improved ATP production capacity.

This is in accord with previous reports by others, including us, showing bezafibrate to be beneficial for certain mitochondrial diseases. This could possibly be attributed to increased ability to utilize fatty acids [29,31,32,34,41].

Furthermore, bezafibrate had a positive effect on mitochondrial morphology, leading to a decrease in mitochondrial elongated phenotype, approaching that of controls (higher levels of solitary mitochondria and lower levels of elongated ones) in the patient fibroblasts. Normalization of mitochondrial morphology and viability upon bezafibrate supplementation was also observed in HFF-1 cells transformed with the mutant p.G362S DNM1L. These results indicate that bezafibrate has an additional role as a regulator of other factors that participate, directly or indirectly, in additional mitochondrial processes, such as the mitochondrial fission/fusion process and quality control. The precise role of bezafibrate in theses.

Nevertheless, the positive effects of bezafibrate could come with a cost, namely increased ROS production, as we observed in this study and our previous findings in some mitochondrial disease cells, albeit the elevations were mild and other cells showed decreased ROS [33,34]. Nevertheless, we think that the elevated ROS and MMP observed in this study is an anticipated consequence of increased respiratory chain activity and mitochondrial content. Increased mitochondrial content was observed with the MitoTracker Green stain in the microtiter wells but could not significantly be verified by microscopy. We did not include the evaluation of peroxisomal function in this study since there was no evidence of peroxisomal dysfunction in our patient [10]. To date, few treatments have been suggested for patients suffering from mitochondrial diseases. For the vast majority of patients, the treatment is limited to treating the symptoms, rather than curing the disease. Still, the administration of small molecules could improve some mitochondrial parameters, and possibly alleviate mitochondrial disease symptoms [28,29,30,31,32,42,43].

### 4.2. Limitations

Obviously, our study is limited to only one patient and was conducted in fibroblasts. Moreover, our patient’s fibroblasts were vulnerable and did not survive multiple passages and freezing, hindering additional experiments. Fibroblasts from our other patient [20], harboring a de novo p.V203M mutation, did not survive culturing. Nevertheless, complementary studies in transformed HFF-1 corroborated the normalization of mitochondrial morphology by bezafibrate, although studies in microtiter wells were not possible due to partial transformation efficiency and decreased viability.

Further studies would be needed to show the consistency of the response to bezafibrate, to precisely elucidate its effect on mitochondrial morphology and quality control in additional experimental systems. Unfortunately, we were not able to conduct a clinical trial in our patient using bezafibrate, due to parental noncompliance with the treatment and follow-up.

### 4.3. Conclusions

Despite its limitations, given the various favorable effects in our study, as well as in other mitochondrial diseases, we suggest that bezafibrate could be offered as a treatment option for patients with certain *DNM1L* mutations.

## Supplementary Materials

The following are available online at https://www.mdpi.com/2073-4409/9/2/301/s1, Figure S1: Mitochondrial content, oxygen consumption and cellular acidification rate.
