# Supplementary material for: Bezafibrate Improves Mitochondrial Fission and Function in DNM1L-Deficient Patient Cells
Source: Cells. 2020 Jan 27;9(2):301. doi: 10.3390/cells9020301 (PMC7072316; doi:10.3390/cells9020301)
Supplement: Supplementary file 1 [file cells-09-00301-s001.pdf]

Supplementary Figure S1

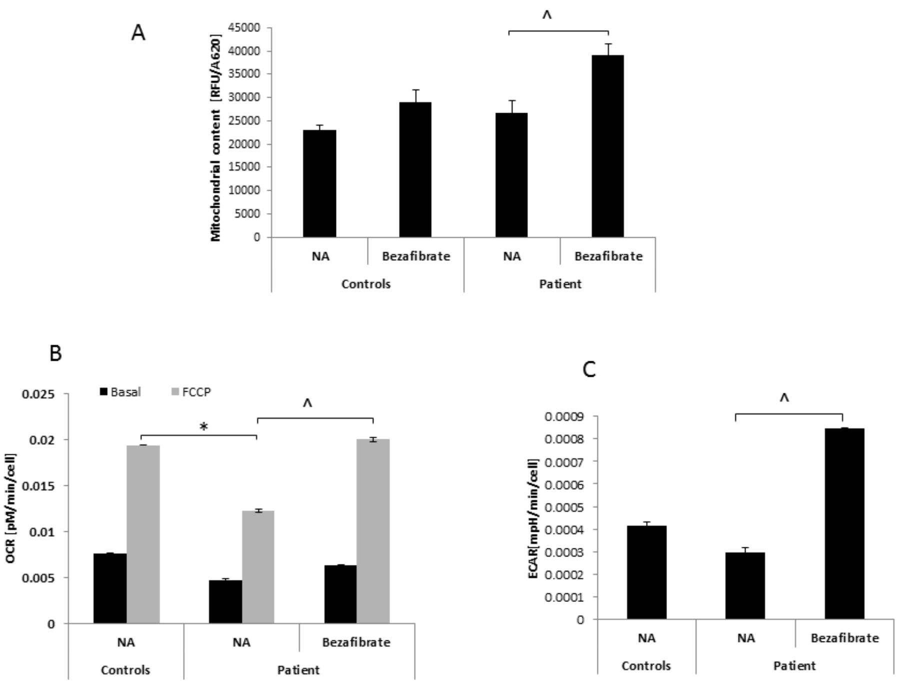

Figure S1. Mitochondrial content, oxygen consumption and cellular acidification rate. Patient and controls fibroblasts were seeded overnight on 96-well plates for mitochondrial content analysis (**A**) or in a XF24 tissue culture plates for OCR (**B**) and ECAR (**C**) measurements. The following day medium was changed and replaced without additive (NA) or in the presence of 100µM Bezafibrate. After 72h (Mitochondrial content was measured with mitotracker green MTG and normalized to cell growth measured with methylene blue MB (**A**). Basal and maximal (FCCP) oxygen consumption rates (OCR) (**B**) and basal extracellular acidification rate (ECAR) (**C**) were measured by XF24 extracellular flux analyzer, and normalized to cellcount. Values are presented mean ± SEM; * $p < 0.05$ patient compared control in corresponding medium; ^ $p < 0.05$ Bezafibrate treated compared to NA .
